# Capsidiol-related genes are highly expressed in response to C*olletotrichum scovillei* during *Capsicum annuum* fruit development stages

**DOI:** 10.1038/s41598-020-68949-5

**Published:** 2020-07-21

**Authors:** Viviane Y. Baba, Adrian F. Powell, Suzana T. Ivamoto-Suzuki, Luiz F. P. Pereira, André L. L. Vanzela, Renata M. Giacomin, Susan R. Strickler, Lukas A. Mueller, Rosana Rodrigues, Leandro S. A. Gonçalves

**Affiliations:** 1 Laboratório de Ecofisiologia e Biotecnologia Agrícola, Programa de Pós-Graduação em Agronomia, Universidade Estadual de Londrina, Londrina, Brazil; 2 Boyce Thompson Institute, Ithaca, USA; 3 Instituto de Biociências, Universidade Estadual Paulista, Rio Claro, Brazil; 4 Empresa Brasileira de Pesquisa Agropecuária, Brasília, Brazil; 5 Laboratório de Citogenética e Diversidade Vegetal, Universidade Estadual de Londrina, Londrina, Brazil; 6 Genética e Melhoramento de Plantas, Universidade Estadual do Norte Fluminense Darcy Ribeiro, Campos dos Goytacazes, Brazil

**Keywords:** Agricultural genetics, Plant breeding, Transcriptomics

## Abstract

*Capsicum annuum* is one of the most important horticultural crops worldwide. Anthracnose disease (*Colletotrichum* spp.) is a major constraint for chili production, causing substantial losses. Capsidiol is a sesquiterpene phytoalexin present in pepper fruits that can enhance plant resistance. The genetic mechanisms involved in capisidiol biosynthesis are still poorly understood. In this study, a 3′ RNA sequencing approach was used to develop the transcriptional profile dataset of *C. annuum* genes in unripe (UF) and ripe fruits (RF) in response to *C. scovillei* infection. Results showed 4,845 upregulated and 4,720 downregulated genes in UF, and 2,560 upregulated and 1,762 downregulated genes in RF under fungus inoculation. Four capsidiol-related genes were selected for RT-qPCR analysis, two 5-epi-aristolochene synthase (*CA12g05030*, *CA02g09520)* and two 5-epi-aristolochene-1,3-dihydroxylase genes (*CA12g05070*, *CA01g05990*). *CA12g05030* and *CA01g05990* genes showed an early response to fungus infection in RF (24 h post-inoculation—HPI), being 68-fold and 53-fold more expressed at 96 HPI, respectively. In UF, all genes showed a late response, especially *CA12g05030*, which was 700-fold more expressed at 96 HPI compared to control plants. We are proving here the first high-throughput expression dataset of pepper fruits in response to anthracnose disease in order to contribute for future pepper breeding programs.

## Introduction

Chili peppers originate from the Americas and they are among the oldest cultivated plants, dating from before 3400 BC. *Capsicum annuum* L. is the most commercially important species, due to its usefulness in the human diet, medicines, beverages, and as ornamentals. Pepper fruits have several nutraceutical benefits for human health, due to a variety of antioxidant, anti-inflammatory, antimicrobial, anti-carcinogenic, and cardio-protective properties^1^.

Anthracnose is caused by *Colletotrichum* spp., and represents the major disease of chili fruit worldwide, leading to significant postharvest yield loss and reducing marketability^2^. Twenty-four *Colletotrichum* species have been identified as pathogens of chili anthracnose, with the three main pathogenic species being *C. scovillei* (previously identified as *C. acutatum*)*, C. truncatum* (syn. *C. capsici*)*, and C. siamense* (previously identified as *C. gloeosporioides*)^3^.

*Colletotrichum* species are able to infect many other parts of the chili plant and the disease has a complex etiology^4,5^, mostly associated with the acutatum, truncatum, and gloeosporioides complexes^3^. *Colletotrichum* species tend to engage in distinct strategies during fruit development stages. In unripe fruit, for instance, there is appressoria formation, hyphal penetration, followed by a quiescence phase, while ripening fruits trigger active infection and colonization. For example, in white strawberry fruits, *C. acutatum* is quiescent and forms appressoria, but it engages in necrotrophic colonization in red fruits^6^. Similar distinct processes of infection and related transcriptional responses have been observed in tomato fruit^7^.

A vast array of chemical compounds play roles in plant defense strategies such as direct defense using toxins and indirect defenses, mediated by phenolic compounds, alkaloids, and terpenoids^8^. The levels of these biochemical compounds can vary according to pepper fruit development stages^9^. One of these compounds is capsidiol, a sesquiterpenoid which has already been described as being related to antifungal activity against *C. gloeosporioides* in pepper fruits^9^. Capsidiol production generally occurs around the infection site of the pathogen, forming a chemical barrier, and plays a defensive mechanism against pathogen interaction^9,10^. Two key enzymes are responsible for capsidiol biosynthesis: 5-epi-aristolochene synthase (*EAS*) and 5-epi-aristolochene dihydroxylase (*EAH*)^9,11^. Park et al.^9^ found *EAS* was significantly induced in ripe fruits infected with *C. gloeosporioides*, and there was a negative relation between the capsidiol level and fruits lesion size.

Given the great importance of healthy pepper fruits in the production, transport, and consumption sectors, investigations on the transcriptional changes during pepper fruit development have advanced in the last years^12,13^. However, the specific effects of anthracnose interaction on transcriptome-level responses are still poorly understood. While studies of individual expression of defense-related genes have provided insights into pepper responses to anthracnose^14,15^, large scale transcriptome studies allow for contrasts of whole expression profiles. These comparisons are an interesting subject for pepper breeding purposes, since anthracnose is caused mainly by *C. scovillei* and can affect unripe and ripe fruits stages, although red pepper fruits seem to be more resistant than green fruits^16,17^.

Our main goal was to elucidate the distinct pepper transcriptional responses to anthracnose in ripe and unripe fruits by studying metabolic pathways using 3′ RNA sequencing (RNA-Seq) and developing a panel of candidate genes for future pepper breeding programs. In addition, we analyzed the transcriptional activity of capsidiol-related genes (*CaEAS* and *CaEAH*) at both fruit development stages under *C. scovillei* infection. The digital gene expression pattern of *CaEAS* (*CA12g05030* and *CA02g09520*) and *CaEAH* (*CA12g05070* and *CA01g05990*) was verified by real-time quantitative polymerase chain reaction (RT-qPCR). This *C. annuum* 3′ transcriptome data constitutes an important high-throughput dataset of distinct transcriptional responses to anthracnose and provides important clues to identify candidate genes related to several pepper metabolic pathways that could be relevant for improvement of pepper resistance against *C. scovillei* in the future. Furthermore, the results will provide a basis to develop better strategies for pepper breeding focusing on anthracnose disease control.

## Results

### Transcriptome sequencing and data mining

The estimate of transcriptional activity of genes under anthracnose infection revealed a high proportion of associated filtered reads (362,449,581—94%), and uniquely mapped reads (283,656,019—77%) in unripe and ripe fruits when data were mapped against the *C. annuum* reference genome (Table 1).

**Table 1:** Summary of sequencing, sequence pre-processing and alignment of reads to the reference *C. annuum* genome, using QuantSeq sequencing, for 48 libraries in unripe and ripe pepper fruits inoculated with *C. scovillei* and mock-inoculated at 24, 48, 72, and 96 h post-inoculation.

Treatment	Inoculation	Time post-inoculation (h)	Repeat	Raw reads	High-quality reads	% of high-quality reads	Uniquely mapped reads	% mapped reads	% feature reads
Unripe	Mock	24	1	12.990.889	12.184.527	94	9.584.189	79	63
			2	3.966.942	3.699.549	93	2.889.847	78	63
			3	14.606.216	13.862.879	95	11.085.840	80	64
		48	1	12.995.915	12.130.332	93	9.418.354	78	63
			2	6.933.438	6.483.410	94	5.120.107	79	65
			3	12.525.732	11.858.690	95	9.241.647	78	64
		72	1	7.424.660	7.009.182	94	5.419.311	77	65
			2	7.479.304	7.054.619	94	5.468.677	78	65
			3	9.634.301	9.028.962	94	7.127.772	79	66
		96	1	13.181.365	12.285.148	93	9.650.275	79	66
			2	10.527.138	9.682.324	92	7.691.708	79	67
			3	6.523.917	6.059.695	93	4.745.300	78	66
	Inoculated	24	1	9.850.667	9.198.742	93	7.080.806	77	62
			2	6.827.939	6.508.457	95	5.112.657	79	64
			3	12.611.459	11.800.483	94	9.387.955	80	65
		48	1	7.627.569	7.215.392	95	5.744.296	80	65
			2	9.035.442	8.589.423	95	6.844.742	80	65
			3	10.053.727	9.431.511	94	7.505.322	80	65
		72	1	6.154.448	5.786.816	94	4.399.277	76	63
			2	3.734.928	3.534.320	95	2.710.166	77	64
			3	4.443.351	4.183.856	94	3.276.723	78	65
		96	1	5.386.086	5.030.122	93	2.317.923	46	65
			2	3.554.552	3.271.355	92	1.573.903	48	66
			3	7.048.073	6.563.789	93	2.771.888	42	65
Average per unripe samples				8.546.586	8.018.899	94	6.090.362	74	65
Ripe	Mock	24	1	8.830.408	8.342.243	94	6.827.228	82	67
			2	12.485.224	11.782.806	94	9.628.905	82	66
			3	9.733.605	9.063.522	93	7.485.642	83	68
		48	1	5.781.929	5.432.891	94	4.256.856	78	68
			2	7.052.114	6.672.680	95	5.591.035	84	66
			3	12.456.326	11.779.954	95	9.928.591	84	67
		72	1	11.630.104	10.681.045	92	8.518.160	80	62
			2	5.431.606	4.961.725	91	3.924.629	79	62
			3	6.594.686	6.104.440	93	4.859.535	80	61
		96	1	9.650.320	9.167.362	95	7.558.657	82	66
			2	8.751.650	8.288.542	95	6.808.386	82	67
			3	9.525.509	9.034.162	95	7.528.533	83	66
	Inoculated	24	1	4.264.122	4.026.402	94	3.349.044	83	66
			2	4.531.714	4.282.299	94	3.527.047	82	67
			3	7.987.388	7.608.792	95	6.381.044	84	67
		48	1	4.688.392	4.365.719	93	3.484.262	80	65
			2	6.353.887	5.982.699	94	4.962.403	83	66
			3	7.389.005	6.990.993	95	5.799.937	83	66
		72	1	5.355.723	4.910.216	92	3.733.959	76	58
			2	6.281.458	5.610.643	89	4.337.099	77	61
			3	6.021.957	5.645.650	94	4.470.158	79	61
		96	1	4.803.738	4.575.877	95	3.385.361	74	65
			2	7.213.368	6.829.997	95	5.324.138	78	67
			3	8.401.214	7.855.339	94	5.816.725	74	68
Average per ripe samples				7.550.644	7.083.167	94	5.728.639	81	65
Total				386.333.505	362.449.581		283.656.019		

### Transcriptome samples clustering analysis

Clustering distance was evaluated for all 3′ RNA-seq pepper samples using PCA and heatmap analysis. In the principal component analysis, components 1 and 2 explained 90% of data variance for treatment (mock vs. inoculated), stage (unripe vs. ripe), and time post-inoculation (24, 48, 72 and 96 h) (see Supplementary Fig. S1).

We observed two distinct clusters for unripe and ripe fruits showing potential differences in the response to anthracnose (see Supplementary Fig. S1A). Ripe fruits showed distinct clusters for mock and inoculated treatment, while unripe fruits showed clusters for early (24 and 48 HPI) and later (72 and 96 HPI) times post-inoculation. Mock and inoculated samples at 24 and 48 HPI showed greater initial transcriptional responses in ripe fruits. Unripe fruits showed greater responses at 72 and 96 HPI for inoculated samples. Ripe fruits showed greater response at 96 HPI (see Supplementary Fig. S1B), in which the degree of response to fungal inoculation was clearly greater than other post-inoculation times. Also, we identified a cluster with all mock and inoculated samples at 24 and 48 HPI for unripe fruits. Biological replicates showed no sample outliers. Heatmap analysis showed concordance with PCA analysis for unripe and ripe fruits (see Supplementary Fig. S1C). Clusters were observed for fruit development stages based on the inoculation treatment for ripe fruits and based on time post-inoculation for unripe fruits. Three biological unripe replicates at 96 HPI were the most distinct from the other samples.

### Differential gene expression profiles among fruit development stages in response to fungal interaction

A panel of statistically significant DEGs (FDR < 0.05) were obtained using DESeq2 analysis in response to anthracnose. The Venn diagram of *C. annuum* transcripts for each fruit development stage showed an overlap between unripe and ripe fruits (1,539), but also revealed distinct stage-specific expression, in which 2,281 DEGs were unique to unripe pepper fruits, while 1,283 transcripts were unique to ripe fruits (see Supplementary Fig. S2). In this way, there were a higher number of regulated genes specific to the unripe and ripe pepper fruits under fungal interaction.

**Figure 1:**
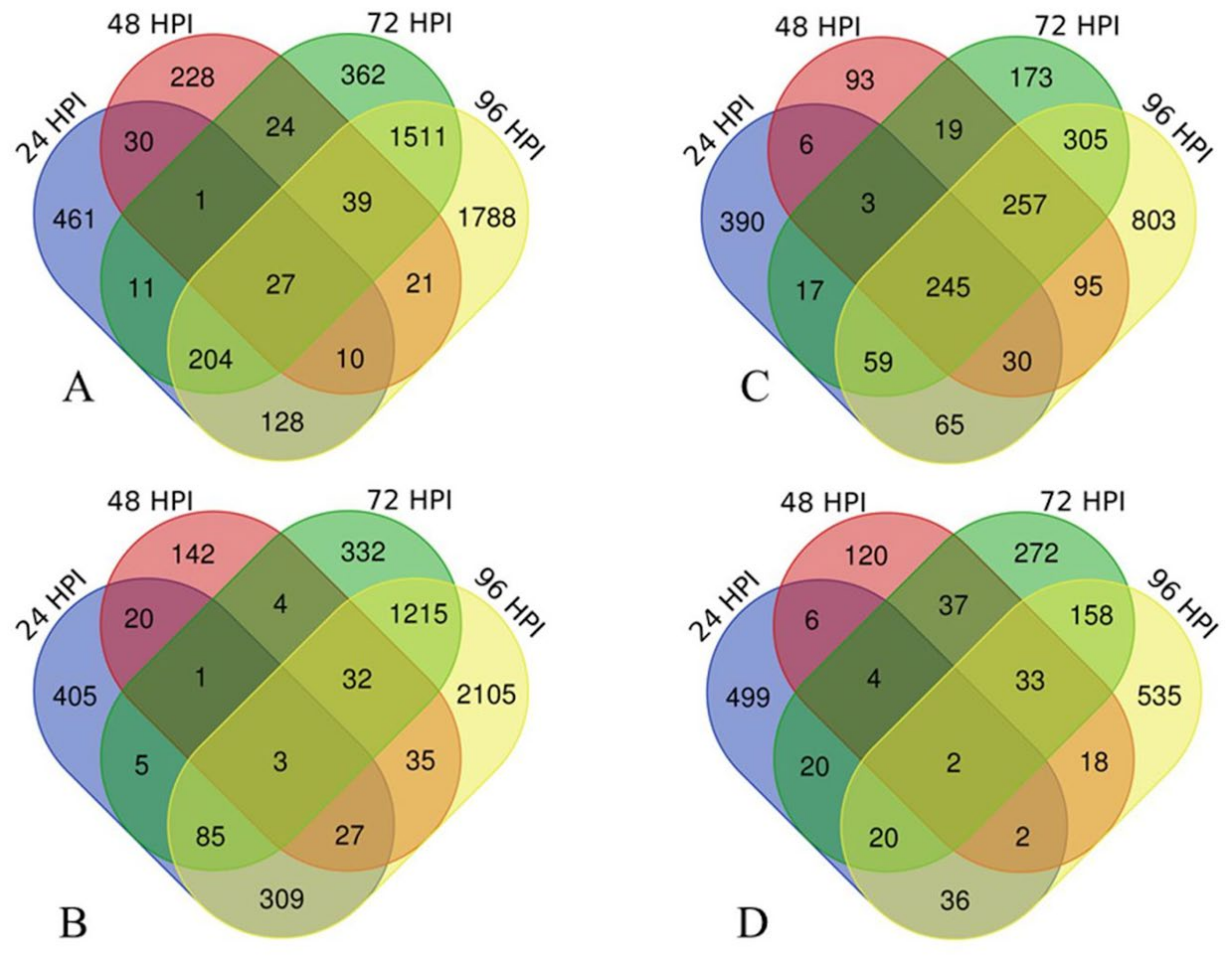
Venn diagrams of differentially expressed genes (DEGs) for inoculation (mock × inoc) in unripe (left) and ripe (right) pepper fruit tissues. Genes up (**A**) and downregulated (**B**) in unripe fruit inoculated with *C. scovillei* (24, 48, 72, and 96 HPI). Genes up (**C**) and downregulated (**D**) in ripe fruit inoculated with *C. scovillei* (24, 48, 72 and 96 HPI). Note that the highest number of unique DEGs was observed at 96 HPI for both unripe and ripe fruits. In addition, unripe fruits also showed high number of unique DEGs at 72 HPI. The number of DEGs showed little overlap at each time post-inoculation, indicating high numbers of distinct transcripts for each fruit development stage.

We also performed transcript abundance analysis using edgeR to identify genes that were significantly (FDR < 0.05) up (Fig. 1A) and downregulated (Fig. 1B) from both fruit stages and at each time post-inoculation (24 to 96 HPI). A different gene expression profile was observed in response to fungal interaction at each time-point analyzed. The highest total number of upregulated genes was found in unripe (4,845) compared with ripe fruits (2,560) (Fig. 1A,C). A similar result was observed for downregulated genes, in which 4,720 genes were observed for unripe and 1,762 for ripe fruits (Fig. 1B,D).

The total number of genes at 72 and 96 HPI in unripe fruits increased more than twofold in relation to ripe fruits in the same time post-inoculation. The highest number of unique DEGs were observed at 96 HPI for both fruit development stages (Fig. 1). However, unripe fruits showed more down (2,105) than upregulated (1,788) genes at 96 HPI (Fig. 1A,B). The opposite occurred for ripe fruits in which the number of unique genes was higher for up (803) than downregulated (535) at 96 HPI (Fig. 1C,D).

The number of up and downregulated unique genes at 24, 48, and 72 HPI showed the opposite profile. Unripe fruits showed more upregulated (461, 228, and 362, respectively) than downregulated unique genes (405, 142, and 332, respectively) (Fig. 1A,B). On the other hand, ripe fruits revealed more downregulated (499, 120, and 272, respectively) than upregulated unique genes (390, 93, and 173, respectively) (Fig. 1C,D). In general, the number of DEGs showed little overlap at each time post-inoculation, indicating high numbers of distinct transcripts for each fruit development stage, except for 72 and 96 HPI up and downregulated unripe genes.

The top 10 differentially expressed genes for each time point post-inoculation in unripe and ripe fruits can be seem in the Table 2. Ripe fruits showed defense response genes at all post-inoculation times, including binding protein, resistance protein, pathogenesis-related protein, pepper esterase, ethylene response factor, cytochrome P450, fatty acid, 5-epi-aristolochene synthase (*EAS*), and 5-epi-aristolochene 1,3-dihydroxylase (*EAH*) genes. Seven candidate genes for capsidiol biosynthesis were also observed in this list (Table 2): three for *EAS* (*CA02g09520, CA12g05030, CA12g05260*) and four for *EAH* (*CA01g05990, CA02g09570, CA12g05070, CA12g05140*).

**Table 2:** List of the top 10 upregulated genes for each time point post-inoculation in unripe and ripe fruits.

Gene ID	Annotation	FDR	
**UNRIPE**			
24 HPI			
*CA09g18430*	Unknown protein	1.06E-94	
*CA02g28000*	Detected protein of unknown function	4.68E-85	
*CA04g04080*	Phytoene synthase	2.31E-83	
*CA11g18070*	Serine carboxypeptidase III	9.24E-70	
*CA04g21250*	Detected protein of confused Function	4.16E-69	
*CA07g15720*	CASP-like protein VIT_01s0010g01870-like	3.67E-60	
*CA03g06040*	Cyanidin-3-O-glucoside 2-O-glucuronosyltransferase-like	1.05E-59	
*CA08g13840*	Germin-like protein subfamily 1 member 20	4.00E-54	
*CA10g09450*	Auxin efflux carrier component, auxin transport protein	1.44E-53	
*CA05g02660*	PREDICTED: BURP domain-containing protein 17-like	4.84E-44	
48 HPI			
*CA08g17070*	18.5 kDa class I heat shock protein-like	4.89E-65	
*CA03g08390*	Translocator protein homolog	5.50E-62	
*CA03g30260*	Heat shock protein, putative	1.59E-43	
*CA09g08990*	Glycerol-3-phosphate acyltransferase 6	3.55E-42	
*CA11g18770*	Ripening-related protein grip22	1.40E-38	
*CA02g16190*	Detected protein of unknown function	7.11E-37	
*CA03g21390*	Heat shock protein 26 (Type I)	7.89E-37	
*CA08g07920*	BAG family molecular chaperone regulator 6-like	1.26E-36	
*CA03g27140*	Detected protein of unknown function	8.90E-33	
*CA05g01800*	Universal stress protein MJ0531-like isoform 1	4.10E-32	
72 HPI			
*CA05g04810*	Zeatin O-glucosyltransferase-like	0	
*CA05g04830*	Multiprotein-bridging factor 1c-like	0	
*CA07g11250*	1-aminocyclopropane-1-carboxylic acid oxidase	0	
*CA09g04530*	Ca^2+^-binding protein 1	0	
*CA12g06260*	UDP-glucose:flavonoid 3-O-glucosyltransferase	0	
*CA02g04610*	Tau class glutathione transferase GSTU15	0	
*CA03g03950*	UDP-sugar:glycosyltransferase	0	
CA02g09520*	5-epi-aristolochene synthase	0	
*CA02g22240*	Unknown protein	0	
*CA05g03050*	Cytochrome P450 CYP736A54	0	
96 HPI			
*CA05g03050*	Cytochrome P450 CYP736A54	0.00E + 00	
*CA11g14520*	Cytochrome P450	0.00E + 00	
*CA04g13070*	Pathogen-related protein-like	3.36E-302	
*CA03g35110*	DNA binding protein homolog	1.26E-281	
CA02g09520*	5-epi-aristolochene synthase	3.51E-271	
*CA02g04360*	Ethylene response factor ERF2	1.53E-259	
*CA08g04180*	Omega-6 fatty acid desaturase, endoplasmic reticulum isozyme 2-like	1.67E-253	
*CA07g11250*	1-aminocyclopropane-1-carboxylic acid oxidase	1.01E-252	
*CA12g22670*	Protein ECERIFERUM 1-like	1.79E-248	
*CA02g15780*	Polyphenol oxidase	1.03E-241	
**RIPE**			
24 HPI			
*CA08g18080*	Allene oxide synthase	6.67E-87	
CA02g09570*	5-epi-aristolochene 1,3-dihydroxylase	2.65E-77	
CA12g05070*	5-epi-aristolochene 1,3-dihydroxylase	7.74E-71	
CA01g05990*	5-epi-aristolochene 1,3-dihydroxylase	5.26E-58	
CA12g05030*	5-epi-aristolochene synthase	4.76E-54	
*CA05g20080*	Isopentenyl diphosphate isomerase	8.13E-54	
*CA03g35110*	DNA binding protein homolog	1.68E-51	
CA12g05140*	5-epi-aristolochene 1,3-dihydroxylase	5.71E-50	
CA02g09520*	5-epi-aristolochene synthase	3.81E-48	
*CA02g22240*	Unknown protein	3.81E-48	
48 HPI			
*CA05g17820*	UTP:alpha-D-glucose-1-phosphate uridylyltransferase	1.46E-71	
*CA03g01800*	Pleiotropic drug resistance protein 1-like	2.44E-61	
*CA07g11250*	1-aminocyclopropane-1-carboxylic acid oxidase	6.33E-57	
*CA05g18370*	Unknown protein	5.39E-52	
*CA08g18080*	Allene oxide synthase (Fragment)	1.89E-50	
*CA09g03220*	Pathogenesis-related leaf protein 4-like	5.05E-48	
*CA01g04790*	Invertase	8.82E-46	
*CA04g10620*	Pepper esterase	7.80E-43	
*CA03g04260*	Pathogenesis-related protein STH-2-like	8.56E-43	
*CA02g04360*	Ethylene response factor ERF2	3.20E-42	
72 HPI			
*CA02g15780*	Polyphenol oxidase	1.19E-168	
*CA02g00210*	Carbonic anhydrase	2.59E-133	
*CA03g03950*	UDP-sugar:glycosyltransferase	5.06E-118	
*CA08g10220*	Wound-induced protein WIN2	2.21E-116	
*CA08g18080*	Allene oxide synthase	5.13E-109	
*CA04g10620*	Pepper esterase	4.34E-106	
CA12g05260*	5-epi-aristolochene synthase	3.80E-101	
*CA03g29750*	Em protein H5-like	7.32E-100	
*CA12g05270*	UV-induced sesquiterpene cyclase	5.43E-96	
CA01g05990*	5-epi-aristolochene 1,3-dihydroxylase	1.49E-95	
96 HPI			
*CA05g04830*	Multiprotein-bridging factor 1c-like	2.68E-153	
*CA05g03050*	Cytochrome P450 CYP736A54	4.58E-144	
*CA02g15780*	Polyphenol oxidase	4.58E-144	
*CA08g18080*	Allene oxide synthase	7.60E-144	
*CA08g04180*	PREDICTED: omega-6 fatty acid desaturase, endoplasmic reticulum Isozyme 2-like	2.83E-143	
*CA11g14520*	Cytochrome P450	2.03E-142	
CA12g05030*	5-epi-aristolochene synthase	3.99E-137	
*CA07g11250*	1-aminocyclopropane-1-carboxylic acid oxidase	5.16E-136	
*CA02g00210*	Carbonic anhydrase	3.25E-135	
CA02g09520*	5-epi-aristolochene synthase	8.02E-133	

*FDR* false discovery rate, *HPI* hours post-inoculation.

*Candidate genes for capsidiol biosynthesis upregulated under *C. scovillei* interaction.

In unripe fruits, the defense response genes were highly expressed, especially at 96 HPI, including *CA02g09520* capsidiol-related genes (Table 2). However, in ripe fruits, we observed more upregulated genes related to capsidiol biosynthesis than in unripe fruits (Table 2). Among the upregulated genes in ripe fruits under pathogen inoculation, we found one *EAS* (*CA02g09529*) and five *EAH* (*CA01g05990, CA02g09570, CA12g05030, CA12g05070*, *CA12g05140*) genes at 24 HPI, one *EAS* (*CA12g05260*) and one *EAH* (*CA01g05990*) gene at 72 HPI (2 genes), and two *EAS* (*CA02g09520*, *CA12g05030*) genes at 96 HPI.

### Transcriptome gene enrichment analysis

In order to verify pepper metabolic pathways that were enriched under anthracnose inoculation, gene enrichment analysis using topGO (p < 0.05) was performed. The dataset for this analysis contained only upregulated genes in response to fungal interaction. We found 32 and 27 descriptive GO terms in the biological processes that were significantly overrepresented under *C. scovillei* for unripe and ripe fruits, respectively. For molecular functions, 48 and 41 descriptive GO terms were significantly enriched for unripe and ripe fruits, respectively (see Supplementary Table S1).

Defense metabolic pathways were enriched in the biological processes level for both fruit development stages, such as defense response to fungus, l-phenylalanine metabolic process, chitin catabolic process and isoprenoid biosynthetic process. Another defense enriched pathway observed for ripe fruits was the ethylene-activated signaling pathway. For molecular function, some of the significant enriched pathways for unripe and ripe fruits were protein serine/threonine kinase activity, related to plant defense response to a pathogen, and chitinase activity, connected with fungus digestion of cell walls, potent inhibitors of fungal growth.

### Top 100 most heterogeneously expressed genes

**Figure 2:**
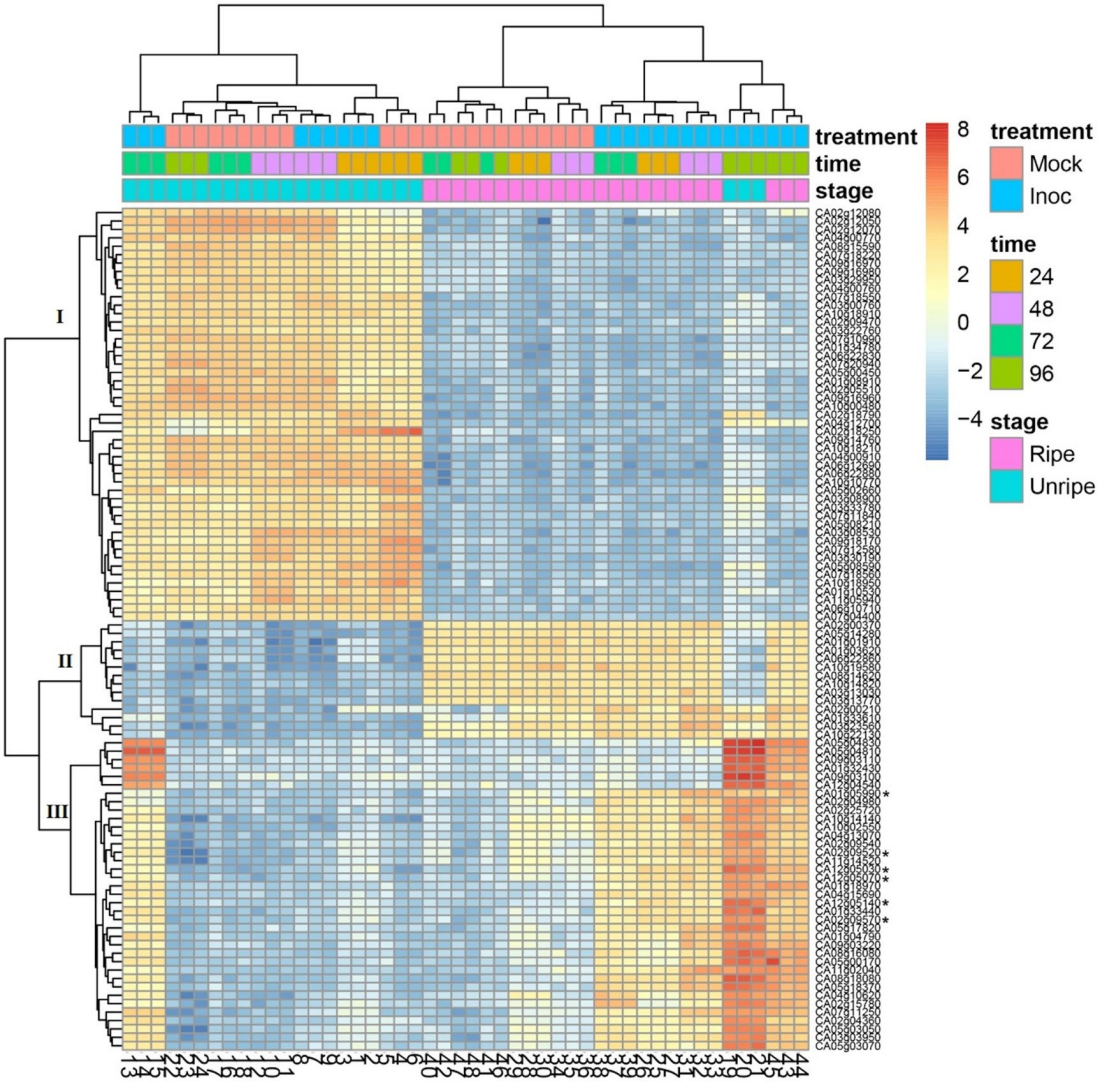
Heatmap analysis representing the transcriptional activity of the 100 most variable genes in unripe and ripe fruits of *C. annuum* after 24, 48, 72, and 96 h post-inoculation with *C. scovillei* and mock inoculation. Rows are genes and columns are samples. Red color indicates high row mean-centered expression levels and blue fields indicate lower row mean-centered expression. Asterisks denote capsidiol-related genes (*CA01g05990*, *CA02g09520*, *CA12g05030*, *CA12g05070*, *CA12g05140*, *CA02g09570*). Note that three well-defined groups of genes were generated in the gene expression profiles. The first group was composed by 49 genes that were induced only for unripe fruits. The second group showed an opposite pattern, where 14 genes were upregulated in ripe fruits. In the third group, 37 genes were upregulated at all time points under *C. scovillei* inoculation in ripe fruits and particularly at 72 and 96 HPI in unripe fruits.

To verify if there was any pattern between up and downregulated genes, a top 100 genes list was produced, including those with the most variable transcription across samples in the 3′ RNA-seq dataset (Fig. 2). Our results allowed us to observe the presence of at least three well-defined groups of genes in the gene expression profiles.

One group was composed of 49 genes that were induced only for unripe fruits, except for inoculated samples at 96 HPI. In general, most of the genes were related to the chlorophyll a/b binding protein that was already described as being related to appressoria formation in pepper-fungal interaction (see Supplementary Table S2). The second group showed an opposite pattern, where 14 genes were upregulated in ripe fruits and downregulated in unripe fruits. In the last group, we observed 37 genes that were downregulated in all mock samples (unripe and ripe fruits) and at early time points (24 and 48 HPI) under fungal interaction in unripe fruits. These same genes were upregulated at all time points under *C. scovillei* inoculation in ripe fruits and particularly at 72 and 96 HPI in unripe fruits.

Interestingly, most of the genes in this group were involved in response to pathogen attack, such as cytochrome P450, pathogen-related protein-like, pepper esterase, and ethylene response factor (see Supplementary Table S2). Six candidate genes related to capsidiol biosynthesis were identified in this group: three *EAS* genes (*CA01g05990*, *CA02g09520*, *CA12g0503*) and three *EAH* genes (*CA12g05070*, *CA12g05140*, *CA02g09570*). These genes were upregulated under fungal interaction for both fruit development stages (unripe and ripe), especially at 96 HPI. For this transcriptome study, these capsidiol-related genes were considered good candidate genes for capsidiol biosynthesis.

### Identification and annotation of capsidiol biosynthesis-related candidate genes

A manual identification and annotation of all capsidiol candidate genes, 5-epi-aristolochene synthase (*CaEAS*) and 5-epi-aristolochene 1,3-dihydroxylase (*CaEAH*) in this transcriptome dataset was produced (Table 3). Results showed that some were incorrectly annotated in the *C. annuum* cv. CM334 (Criollo de Morelos 334) genome data. Eleven *EAS* and 14 *EAH* genes showed high e-values (0.0) and scores above 500. In addition, all candidate genes presented the specific conserved domain in their protein sequences, pfam03936 (*EAS*) and pfam00067 (*EAH*), both already described in other plants.

**Table 3:** Description of annotated *C. annuum* candidate genes related to capsidiol biosynthesis.

Gene ID	Acession number	Manual annotation	Genome annotation	E-value	Score	Protein size	Conserved domain
*CA12g05020*	O65323.1	5-epiaristolochene synthase	Vetispiradiene synthase	0.0	1,154	559	pfam03936
*CA12g05150*	O65323.1	5-epiaristolochene synthase	Vetispiradiene synthase	0.0	1,051	559	pfam03936
*CA12g05060*	O65323.1	5-epiaristolochene synthase	UV-induced sesquiterpene cyclase	0.0	1,050	563	pfam03936
CA12g05030*	O65323.1	5-epiaristolochene synthase	5-epi-aristolochene synthase	0.0	1,050	559	pfam03936
CA02g09520*	O65323.1	5-epiaristolochene synthase	UV-induced sesquiterpene cyclase	0.0	1,011	563	pfam03936
*CA08g05300*	O65323.1	5-epiaristolochene synthase	UV-induced sesquiterpene cyclase	0.0	827	472	pfam03936
*CA12g05310*	O65323.1	5-epiaristolochene synthase	Vetispiradiene synthase	0.0	816	510	pfam03936
*CA12g05260*	O65323.1	5-epiaristolochene synthase	5-epi-aristolochene synthase	0.0	635	382	pfam03936
*CA12g05170*	O65323.1	5-epiaristolochene synthase	Viridiflorene synthase-like	0.0	609	379	pfam03936
*CA12g09360*	O65323.1	5-epiaristolochene synthase	Terpene synthase	0.0	565	552	pfam03936
*CA12g09250*	O65323.1	5-epiaristolochene synthase	Terpene synthase	0.0	524	481	pfam03936
CA01g05990*	Q94FM7.2	5-epiaristolochene 1,3-dihydroxylase	CYP71D51v2	0.0	830	515	pfam00067
CA12g05140*	Q94FM7.2	5-epiaristolochene 1,3-dihydroxylase	Cytochrome P450 71D7-like	0.0	799	501	pfam00067
CA12g05070*	Q94FM7.2	5-epiaristolochene 1,3-dihydroxylase	CYP71D51v2	0.0	769	514	pfam00067
*CA12g05220*	Q94FM7.2	5-epiaristolochene 1,3-dihydroxylase	CYP71D51v2	0.0	761	513	pfam00067
CA02g09570*	Q94FM7.2	5-epiaristolochene 1,3-dihydroxylase	CYP71D51v2	0.0	760	515	pfam00067
*CA01g12720*	Q94FM7.2	5-epiaristolochene 1,3-dihydroxylase	Cytochrome P450	0.0	717	493	pfam00067
*CA01g12560*	Q94FM7.2	5-epiaristolochene 1,3-dihydroxylase	Premnaspirodiene oxygenase-like	0.0	709	495	pfam00067
*CA06g13700*	Q94FM7.2	5-epiaristolochene 1,3-dihydroxylase	CYP71D49v1	0.0	589	496	pfam00067
*CA07g03270*	Q94FM7.2	5-epiaristolochene 1,3-dihydroxylase	CYP71D48v1	0.0	577	493	pfam00067
*CA07g11990*	Q94FM7.2	5-epiaristolochene 1,3-dihydroxylase	CYP71D47v1	0.0	573	498	pfam00067
*CA01g08100*	Q94FM7.2	5-epiaristolochene 1,3-dihydroxylase	CYP71D48v2	0.0	572	504	pfam00067
*CA10g06850*	Q94FM7.2	5-epiaristolochene 1,3-dihydroxylase	Cytochrome P450, putative	0.0	536	502	pfam00067
*CA02g19590*	Q94FM7.2	5-epiaristolochene 1,3-dihydroxylase	Cytochrome P450	0.0	531	509	pfam00067
*CA02g19610*	Q94FM7.2	5-epiaristolochene 1,3-dihydroxylase	Cytochrome P450	0.0	523	514	pfam00067

*Candidate genes for capsidiol biosynthesis upregulated in pepper fruits under *C. scovillei* interaction.

### Transcriptional validation of capsidiol biosynthesis-related candidate genes

**Figure 3:**
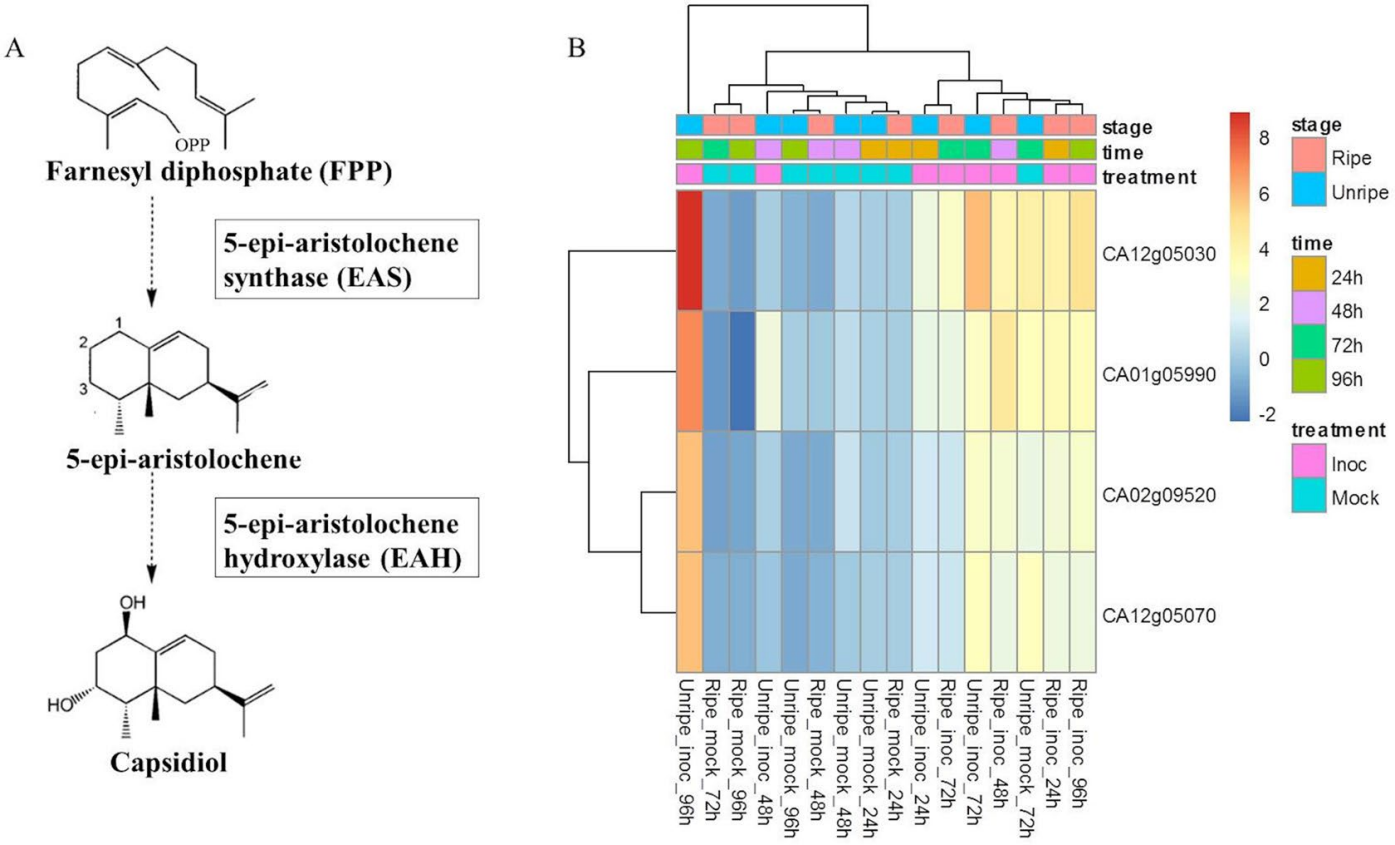
Diagram of capsidiol metabolic pathway (**A**) including capsidiol-related genes expression profile (**B**). Heatmap analysis representing *CaEAS* and *CaEAH* gene expression patterns obtained using RT-qPCR analysis for unripe and ripe fruits of *C. annuum* after 24, 48, 72, and 96 h post-inoculation (HPI) with *C. scovillei* and mock inoculation. Rows are genes and columns are samples. Red color indicates high expression levels and blue fields indicate lower expression. The mean values for *CaEAS* and *CaEAH* relative expression were normalized using *CaEF1α* and *CaUEP*. 24 HPI mock inoculation was set to 1, used as calibrator.

Considering the importance of capsidiol candidate genes to pepper resistance against anthracnose disease and to validate the digital expression profile of the 3′ RNA-Seq data, four candidate genes from *CaEAS* (*CA12g05030*, *CA02g09520*) and *CaEAH* (*CA12g05070*, *CA01g05990*), the key genes in the capsidiol biosynthesis pathway, were selected for RT-qPCR analysis (Fig. 3A). RT-qPCR of capsidiol candidate genes showed stage-specific expression profile consistent to those predicted by 3′ RNA-Seq (Fig. 3B) in the DEG analysis.

It was observed that *CA12g05030* showed a greater number of transcripts in both fruit development stages. Unripe fruits showed a late response to anthracnose but showed the highest expression levels for both capsidiol-related genes (*CaEAS* and *CaEAH*) at 72 and mainly at 96 HPI under *C. scovillei* inoculation (Fig. 3B). In relation to ripe fruits, the abundance of *CaEAS* and *CaEAH* transcripts in response to fungal interaction was rapidly induced starting at 24 HPI (Fig. 3B). Similar to unripe fruits, the transcript peak was detected at 96 HPI.

## Discussion

An overview of *C. annuum* pathways activated in response to development of the fungus *C. scovillei* in unripe and ripe fruits was obtained using pepper transcriptome analyses and a well-annotated genome. According to a previous study^18^, 3′ RNA-Seq is a powerful strategy to detect DEGs and for accurately determining gene expression at a low cost. Transcriptome analysis of fungal-fruit interactions in solanaceous crop plants has previously focused on the traditional method of RNA-Seq. Simultaneous transcriptome analysis of *C. gloeosporioides* and tomato fruit pathosystem, during different stages of infection, revealed the fungal arms strategy and fruit defense response^7^. In some *C. annuum* pathosystems, it has been demonstrated that genes related to resistance response have their transcriptional activity induced, as in the case of *Capsicum chlorosis virus* (*CaCV*)^19^, *Cucumber mosaic virus* (*CMV*)^20^, and three pathogen infections of leaves (*Phytophthora infestans*, *Pepper mottle virus*, and *Tobacco mosaic virus* P0 strain)^13^.

Although global gene expression profiling was performed to elucidate molecular mechanisms either on the pepper fruits related to pungency, fruit repining, abiotic, and biotic stress^12,13,21–26^ or the anthracnose pathogen^27–29^, the mechanisms of pepper fruit defense response against *Colletotrichum* spp. infection using large transcriptome resources are lacking. This is the first expression analysis of *C. scovillei* infected fruit ripening in pepper to provide valuable information on molecular mechanisms. *C. scovillei* was first described in Thailand in 2008^30^, and was also previously reported in Japan, Brazil, China, Korea, and Malaysia^31–38^. *C. scovillei* is considered one of the most widespread and commonly reported *Colletotrichum* species causing anthracnose in chili through-out Southeast Asia and South America^3^.

Pepper transcriptome expression profiles showed different patterns for unripe and ripe fruits and also for mock and inoculated treatments. The highest total number of expressed genes was found in unripe compared to ripe fruits. The different number of DEGs between fruit development stages was expected since unripe and ripe fruits are phenotypically and biochemically different^9^. In a previous analysis of transcriptomes across pepper fruit developmental stages by Martínez-López et al.^12^, distinctive transcriptomic profiles were also observed, where fruits ripening from 40 to 60 DAA were characterized predominantly by a global decrease in gene expression, signaling the end of maturation and the beginning of senescence of chili pepper fruit. Red pepper fruit showed more specialized and less diverse genes^12^.

Although ripe fruits of other plant species are generally susceptible to pathogen infection, pepper fruits revealed distinct responses to the anthracnose. Ripe fruits present higher amounts of some biochemical compounds (e.g. capsidiol) relative to unripe fruits, and those compounds can be positively related to fruit resistance against fungal disease^9,10,39^. Previous studies identifying sources of resistance in *Capsicum* species in response to *Colletotrichum* spp. infection showed that ripe fruits are more resistant to anthracnose compared to unripe fruits^17,40–42^. This could be explained by the rapidly induced expression of *CaEAS* and *CaEAH* genes in response to *C. scovillei* in ripe pepper fruits, while in unripe fruits a late and high upregulation of capsidiol biosynthetic genes were observed. The prompt response of phytoalexin production is more important for the plant defense system than the final concentration accumulated in plant tissue.

A microarray analysis of the interaction of *C. acutatum* with white and red strawberries reveals differences in gene expression possibly related to differing susceptibility and different genes were specifically transcribed only in white or red fruits^6^. During fruit storage and ripening, significant natural physiological changes occur such as tissue extracellular pH, activation of ethylene synthesis and other phytohormones, cuticular changes, cell-wall loosening, increase of soluble sugars, decline of antifungal compounds^28^, and these changes can release the pathogen from its quiescent state and promote a necrotrophic and pathogenic lifestyle^28,43^.

The transcript accumulation in unripe and ripe fruits is dependent on the infection and colonization strategies employed by *Colletotrichum* species, described as hemibiotrophic, which consists of a short biotrophic phase followed by a necrotrophic stage. In unripe fruit, there is formation of appressoria, hyphal penetration and a quiescence phase, while fruit ripening triggers active fungal infection and colonization^6,7,29^. Simultaneous transcriptome analysis of *C. gloeosporioides* and tomato fruit also revealed defense genes induced in stage-specific fungal colonization^7^. Colonization of unripe tomato fruit by *Colletotrichum* initiated defensive responses that limit fungal growth and development, and during fruit ripening, several physiological processes occur that correlate with increased fruit susceptibility^7,43^. This response is different in non-climacteric fruits, which includes pepper fruits.

Plants induce multiple arrays of defense systems against pests and pathogens attack, including a set of preformed structures and inducible reactions^44^. The chemical inducible defense response against pathogen attack involves the activation of defense genes, formation of reactive oxygen species (ROS), synthesis of pathogenesis-related (PR) proteins, localized cell wall reinforcement, and the production of antimicrobial compounds^45^.

Salicylic acid (SA) is associated with resistance to biotrophs and hemibiotrophs, while jasmonic acid (JA) and ethylene (ET) regulate defense during necrotrophic infection^46,47^. The transcript accumulation of JA and ET responsive genes such as plant defensin 1.2 (PDF1.2)*,* Lypoxygenase 3 (Lox3)*,* Allene oxide synthase (AOS), ACC synthase 2 (ACS2), phenylalanine ammonia-lyase 3 (PAL3), and pathogenesis related proteins (PR2 and PR5) were more rapid and had higher induction in the resistant cultivar of chili and *C. truncatum* pathosystem^15^. These genes, related to pepper defense response against fungal interaction, were observed in this study, and were also rapidly induced in ripe fruits, while in unripe fruits the response was delayed.

Another upregulated gene observed in ripe fruits was pepper esterase (*PepEST*), which was already described as being highly expressed in ripe pepper fruits under *C. gloeosporioides* interaction^48^. *PepEST* is involved in the hydrolysis of the external layer of fungal cell walls, leading to inhibition of appressoria formation and activating the defense signaling pathways^49,50^. Resistance in ripe fruits might also be related to the accumulation of ET in non-climacteric pepper fruits and can act as a defense hormone providing resistance to diseases, as the hormone promotes susceptibility in climacteric fruit ripening^43^. According to Oh et al.^51^, non-climacteric fruits show enhanced disease resistance to phytopathogens during ripening. Six genes involved in the defense of the ripe pepper fruit against *C. gloeosporioides* invasion and colonization were induced, including cytochrome P450 protein, esterase, and MADS-box protein^51^. All these genes were also induced in the 3′ RNA-Seq study for ripe fruits. Beyond capsidiol-related genes, significant DEGs cytochrome P450, pathogenesis related proteins were upregulated for ripe fruits (*CA10g02550, CA11g14520, CA05g03050, CA05g03070, CA04g13070, CA03g04260*) and unripe fruits (*CA05g03070, CA03g04260*) in response to *C. scovillei*. However, pepper esterase (*CA04g10620*), allene oxide synthase (*CA08g18080*), and ethylene response factor (*CA02g04360*) were upregulated only for ripe fruits with p-values 2.70E-10, 1.18E-51, and 9.24E-31, respectively.

Capsidiol has been proposed to be an important ‘chemical weapon’ employed by plants in defending against pathogens^52^. Capsidiol is a sesquiterpenoid phytoalexin produced in *Nicotiana* and *Capsicum* species in response to pathogen attack^9,52^. This compound can exhibit fungistatic activity for many fungal species^9,11^ and capsidiol-related genes are considered an important gene involved in pepper tolerance against anthracnose disease. Capsidiol is produced via cyclization of farnesyl pyrophosphate (FPP) to 5-epi-aristolochene by 5-epi-aristolochene synthase (*EAS*), followed by two hydroxylation reactions catalysed by 5-epi-aristolochene dihydroxylase (*EAH*) also known as cytochrome P450 from subfamily CYP71D^11,53^.

Capsidiol-related genes were already described to improve anthracnose resistance in ripe pepper fruits^9^. Lee et al.^10^ showed that a subset of *EAS/EAH* gene family members was highly induced upon *Phytophthora infestans* attack in parallel with capsidiol accumulation. They also suggested that *EAS* and *EAH* genes formed a chemical barrier of nonhost resistance against *P. infestans* in which the fungus could not overcome the toxicity. Song et al.^52^ demonstrated that capsidiol plays an important role in defending against *Alternaria alternata* and *Nicotiana attenuata* pathosystem. The same authors showed that many genes leading to sesquiterpene production were strongly upregulated, including the capsidiol biosynthetic genes. In addition, capsidiol exhibited strong anti-fungal in vitro activity against *A. alternata* and accumulation of capsidiol.

The presence of genes in clusters mentioned by a previous study^10^ and composed of multiple copies of highly induced *EAS/EAH* genes that includes *CA12g05030* (*CaEAS*) and *CA12g05070* (*CaEAH)* was also observed. These gene clusters are located in a 1.3 Mb expanded region of *C. annuum* on chromosome 12 and is composed of four *CaEAS* (*CA12g05020*, *CA12g05030*, *CA12g05060, CA12g05150*) and two *CaEAH* (*CA12g05070*, *CA12g05140*) genes^10^. In addition, the capsidiol biosynthetic pathway is stimulated during the nonhost interaction between pepper and pathogen infection^10^.

Pepper plants, by increasing expression of key capsidiol biosynthesis genes, likely increase the capacity to produce capsidiol during fruit development stages and to accumulate it in ripe fruits. *EAS* was already described as a key enzyme involved in capsidiol biosynthesis and seems to be associated with the enhanced synthesis of capsidiol in response to *C. scovillei* in ripe fruits. The transcriptome dataset produced here can serve as a powerful tool for future analysis of several other metabolic pathways mentioned in this study, in which focused only on capsidiol-related genes. It opens new possibilities to analyze genes that could be important for pepper breeding programs in the future, to improve its resistance against *C. scovillei*.

Our results provide a transcriptome-level overview of the changes in *C. annuum* gene expression profiles under fungal interaction using a pipeline for 3′ RNA-Seq analysis. Overall, the analysis reveals distinct stage-specific gene expression in unripe and ripe pepper fruits in response to the pathogen using genetic mechanisms to produce defense proteins. In particular, we identified and selected capsidiol-related genes to validate their differentially expressed profile by using RT-qPCR analysis. In this way, we generated a reliable panel of up and downregulated candidate genes that can be used in future projects to improve the knowledge about *C. annuum* × *C. scovillei* interactions.

## Material and methods

### Plant material

Seeds of *Capsicum annuum* accession from GBUEL103 (susceptible) and GBUEL104 (resistant to bacterial spot, pepper yellow mosaic virus, and anthracnose^41^) were obtained from the Universidade Estadual de Londrina (UEL) seed germplasm. Samples were sown on a tray with organic plant substrates and, after the emergence of two pairs of true leaves, seedlings were transferred individually to plastic pots containing a mixture of soil and substrate (2:1, w:w ratio). Plants were grown in a greenhouse following practices recommended for pepper cultivation.

### Anthracnose inoculation

Unripe (35 days after anthesis—DAA) and ripe (50 DAA) pepper fruits were detached from the plant and were sterilized in 1% (w/v) sodium hypochlorite solution for five min, followed by three washes with distilled water for one min. A *C. scovillei* spore suspension (1 × 10^6^ conidia/mL^−1^) was prepared with a virulent isolate “8.1” (NCBI accession numbers: MN121780, MN121791, MN121802, MN121811, MN121822). Inoculation was performed under laboratory conditions by the injection method in the central part of the fruit, using a Micro Syringe Model 1705 TLL (Hamilton, Switzerland). The needle depth was fixed at 1 mm to ensure inoculum volume and uniformity of lesion size. Control fruits were similarly treated and processed with distilled water for mock inoculation. Pepper fruits were incubated in the dark for 24 h at 25 °C and were kept in a humid chamber for subsequent 12 h light/dark cycles. Fruits of the two development stages were sampled at 24, 48, 72, and 96 h post-inoculation (HPI). All samples were frozen immediately in liquid nitrogen and stored at − 80 °C until RNA extraction. A susceptible cultivar (GBUEL103) was treated using the same inoculation conditions to validate successful pathogen inoculation in the resistant accession (GBUEL104) (see Supplementary Fig. S3).

### RNA extraction, library construction and sequencing procedures

Total RNA of resistant pepper fruits was extracted using the TRIzol reagent (Thermo Fisher Scientific, Waltham, MA, USA) and purified using the PureLink RNA Mini kit (Thermo Fisher Scientific, Waltham, MA, USA). All the samples were treated with DNase I (RNase-free, Invitrogen, Carlsbad, California, USA). RNA quantity, purity and integrity were verified by spectrophotometry using NanoDrop ND-1000 (Thermo Fisher Scientific, Waltham, MA, USA), Qubit fluorometric quantitation (Thermo Fisher Scientific, Waltham, MA, USA) and Agilent 2100 Bioanalyzer Chip DNA 1000 series II (Agilent Technologies, Santa Clara, California, USA). All reagents were used according to the manufacturer's instructions. Libraries were prepared from *C. annuum* under mock (water) and *C. scovillei* inoculation, including two stages of fruit development (unripe and ripe) at four time points post-inoculation (24, 48, 72 and 96 h) with three biological replicates for each inoculation-by-stage-by-time condition resulting in a total of 48 libraries. Sequencing was performed at the Biotechnology Resource Center, Institute of Biotechnology, Cornell University, Ithaca, NY, USA. For each sample, 2 µg of total RNA was used to prepare mRNA libraries, using the QuantSeq 3′ RNA-Seq kit by Lexogen^54^ to generate sequences close to the 3′ end of polyadenylated RNAs. High-throughput sequencing was performed using the Illumina NextSeq 500 platform, yielding single-end 75 base pair (bp) reads.

### Transcriptome data analysis

The 3′ RNA-Seq data were processed according to the data analysis workflow recommended by Moll et al.^54^. Raw reads were trimmed and filtered for quality and adaptor contamination using BBDuk v37.36 (https://sourceforge.net/projects/bbmap/). The first 12 bp were trimmed from each sequence read. Subsequently, quality trimming of reads was performed using the Phred algorithm, set to Q20. Trimmed reads with a length of less than 35 bp were discarded. FastQC v0.11.5 (www.bioinformatics.babraham.ac.uk/projects/fastqc/) was used to evaluate the quality of reads before and after trimming. Filtered reads were mapped to the pepper reference genome *C. annuum* cv. CM334 v1.55^24^ available at the Sol Genomics Network website^55^ using STAR v2.4.2a^56^. Mapped reads were quantified by HTSeq-count^57^ to obtain digital gene expression read counts from uniquely aligned reads. In order to adequately capture reads mapping to 3′ ends, the GTF file was modified to include 300 bp extensions after the coding sequences (CDS) using the BEDTools *slop* function^58,59^ to increase the size of each feature in the file; this extension length was used since it minimized “no feature reads” to the greatest degree possible while maintaining a relatively low number of “ambiguous reads.”

### Differentially expressed genes

Analysis of differentially expressed genes (DEGs) was performed for both fruit development stages in response to anthracnose infection. The DEGs (FDR < 0.05) were determined for pairwise comparisons between mock and inoculated samples, and they were analyzed in two different ways: (1) using DESeq2^60^ comparing unripe and ripe fruits; (2) using edgeR^61^ at each time point (24, 48, 72, and 96 HPI) in unripe and ripe fruits. For DEG analysis using DESeq2 and edgeR, library size normalization was conducted using the calcNormFactors function in edgeR and accounted for using sample-specific scaling factors in the DESeq function of DESeq2. In addition, we annotated the top 10 upregulated genes at each time point analyzed. All samples were included in a principal component analysis (PCA) and hierarchical clustering heatmap analyses using DESeq2. Venn diagrams were developed using Calculate and Draw custom Venn Diagrams (https://bioinformatics.psb.ugent.be/webtools/Venn/). Gene ontology (GO) overrepresentation analyses for upregulated genes in response to fungal interaction of unripe and ripe fruits were performed using topGO R package (p < 0.05)^62^. Additional hierarchical clustering of the 100 most variable expressed genes across the samples was conducted using the pheatmap function^63^. For hierarchical clustering, Euclidean distances were calculated from data transformed using the rlog() function and mean centered. All DEG analyses were performed in R^64^.

### Identification and annotation of capsidiol biosynthesis-related genes

Protein coding sequences of 5-epi-aristolochene synthase (NCBI accession number: O65323.1) and 5-epi-aristolochene 1,3-dihydroxylase (NCBI accession number: Q94FM7.2) genes previously described in plants^11,65^ were used as query sequences to search for their respective orthologs in our pepper transcriptome dataset. A manual annotation was performed for capsidiol biosynthesis-related genes in the *C. annuum* transcriptome using tBLASTn at NCBI^66^ with the UniProtKB and Swissprot databases and BLASTn at the Sol Genomics Network for pepper databases^24^. We used a cutoff of 500 for minimum bit score, as well as requiring the presence of the conserved domain (pfam03936 and pfam00067) in the protein sequence to manually annotate capsidiol-related genes.

### RT-qPCR transcriptional validation

Primers from capsidiol-related genes (*CaEAS* and *CaEAH*) were designed using CLC Genomics Workbench v.9.5.3 (https://www.qiagenbioinformatics.com/) to amplify nucleotide sequences ranging from 100 to 207 bp with annealing Tm of 55 °C ± 2 °C (see Supplementary Table S3). Complementary DNAs (cDNAs) of all samples were synthesized using GoScript Reverse Transcription System Kit (Promega, Madison, Wisconsin, USA), following the manufacturer’s instructions, in a final volume of 20 µL and using 2.5 µg of total RNA.

Transcriptional profiles of genes were analyzed using ViiA 7 Real-Time PCR System (Thermo Fisher Scientific, Waltham, MA, USA) equipment. The reactions consisted of a total volume of 15 μL with 7.5 μL of GoTaq qPCR Master Mix (Promega, Madison, Wisconsin, USA), 0.5 μL of forward and reverse primer (10 μM), 1 μL of cDNA (25 ng μL^−1^), and 5.5 μL of nuclease-free water. The amplification conditions were 94 °C for 5 min, followed by 40 cycles of 94 °C for 30 s, 55 °C for 45 s and 72 °C for 30 s, followed by melting curve analysis to verify the presence of a single RT-qPCR product. All reactions were performed with three biological replicates and followed MIQE guidelines for RT-qPCR experiments^67^.

Relative expression levels of capsidiol-related genes were analyzed by GenEx 6.1 software (MultiD Analyses AB, Göteborg, Sweden) according to the default parameters. Gene normalization analysis was performed using *CaEF1α* and *CaUEP* gene expression profiles as reference genes^68^. The value 1 was assigned to the library 24 HPI mock inoculation from unripe and ripe fruits, as calibrator samples. The amplification efficiency was calculated using LinRegPCR^69^ (see Supplementary Table S3). The heatmap of *C. annuum* genes’ transcriptional activities was generated in R^64^ using the pheatmap package^63^.

## Supplementary information

Supplementary information
