# Supplementary material for: Capsidiol-related genes are highly expressed in response to Colletotrichum scovillei during Capsicum annuum fruit development stages
Source: Sci Rep. 2020 Jul 21;10:12048. doi: 10.1038/s41598-020-68949-5 (PMC7374708; doi:10.1038/s41598-020-68949-5)
Supplement: Supplementary file 1 — Supplementary information [file 41598_2020_68949_MOESM1_ESM.pdf]

Supplementary information file

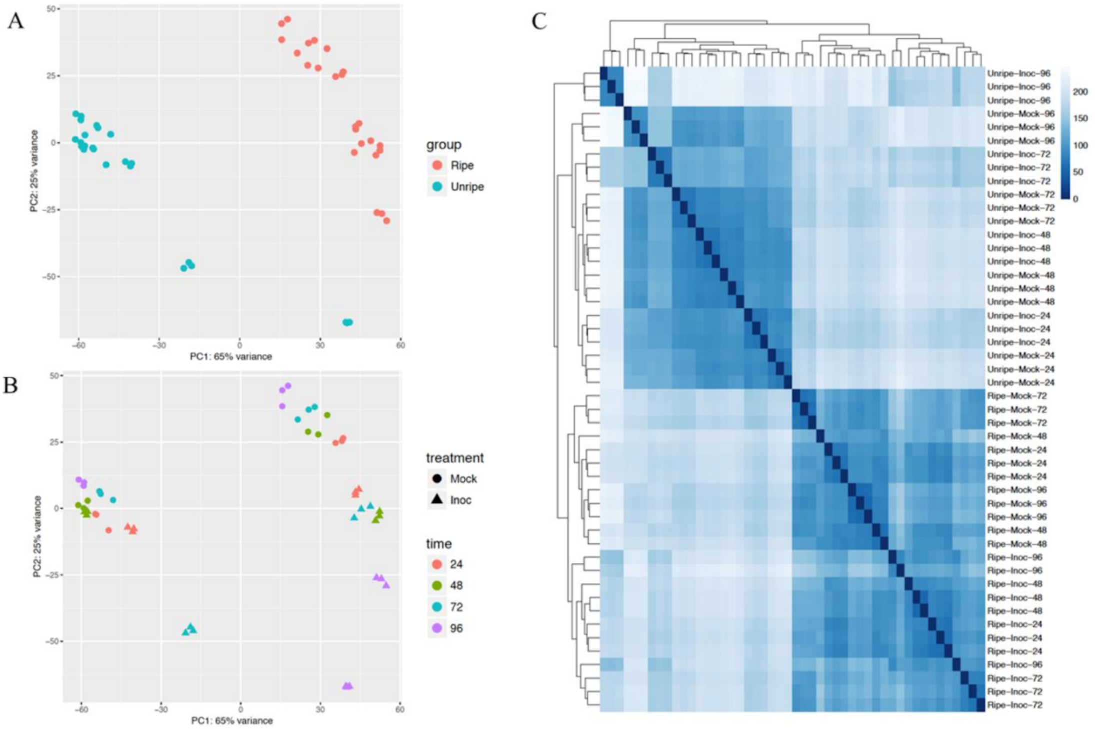

Supplementary Figure 1. Pepper transcriptome clustering analysis among 48 libraries. PCA analysis for fruit development stages: unripe (blue color) and ripe (red color) (A); and for inoculated with *C. scovillei* (inoc - triangle) and with water (mock - circles) and time post-inoculation (24, 48, 72, and 96 h) comparisons (B). Heatmap analysis of cluster distance for all pepper samples (C). The intensity of blue color is proportional to the similarities between samples. Note that in both analyses (PCA and heatmap) distinct clusters were generated based on fruit development stages, inoculation treatment, and post-inoculation time. Unripe fruits showed greater responses at 72 and 96 h for inoculated samples, as well as for ripe fruits at 96 h. Biological replicates showed no sample outliers.

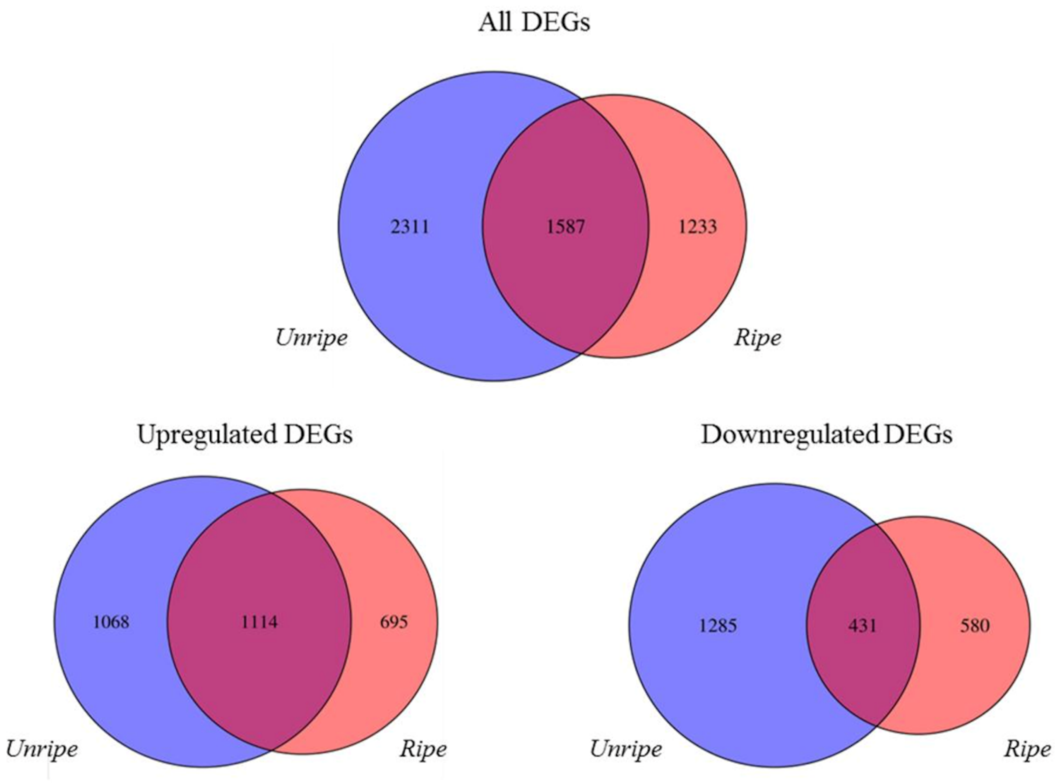

Supplementary Figure 2. Venn diagram of differentially expressed genes (DEGs) for inoculation (mock x inoc) from unripe (blue) and ripe (red) pepper fruit transcriptomes in response to *C. scovillei*. Note that high numbers of distinct stage-specific transcripts were observed for each fruit development stage. The higher number of unique DEGs was observed for unripe fruits compared to ripe.

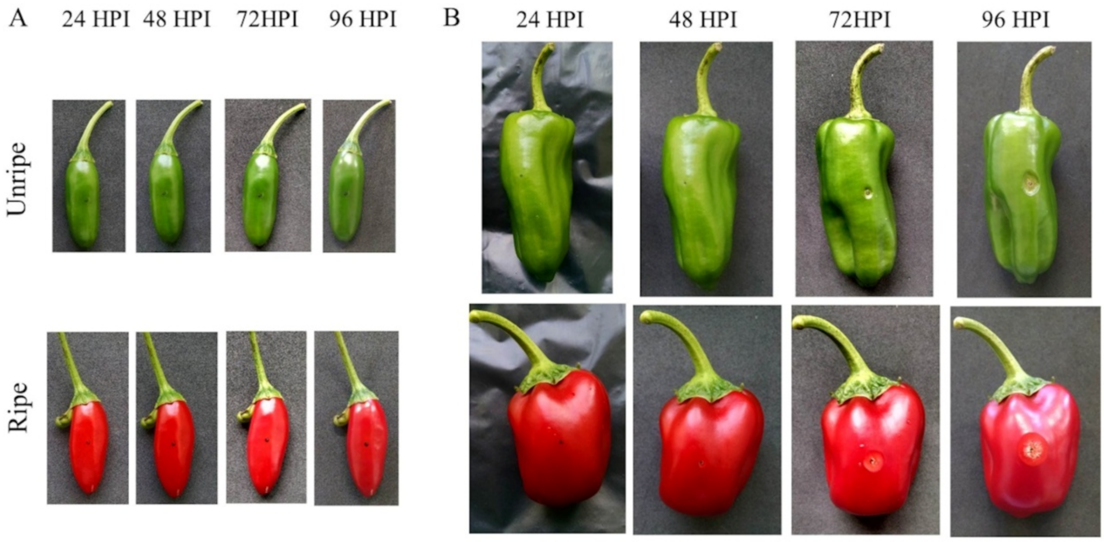

Supplementary Figure 3. *C. scovillei* inoculation in unripe (35 DAA) and ripe pepper fruits (50 DAA) of the resistant accession GBUEL104 (A) and susceptible accession GBUEL103 (B). Photographs were taken at 24, 48, 72, and 96 hours post-inoculation (HPI). Note that the visual anthracnose symptoms appeared in unripe and ripe susceptible pepper fruits at 72 HPI (B), while no symptoms developed on resistant pepper fruits throughout the experiment (A). The fungal isolate produced the typically necrotic, sunken anthracnose symptom at 96 HPI on the susceptible accession (B).

Supplementary Table 1. Enriched GO terms of upregulated genes in unripe and ripe fruits in response to *C. scovillei* inoculation.

GO ID	Term	p-value
**UNRIPE**		
**Biological Process**		
GO:0009070	serine family amino acid biosynthetic process	8.10E-06
GO:0006534	cysteine metabolic process	5.00E-05
GO:0016567	protein ubiquitination	7.30E-05
GO:0006563	L-serine metabolic process	0.00011
GO:0009073	aromatic amino acid family biosynthetic process	0.00021
GO:0050832	defense response to fungus	0.00022
GO:0042742	defense response to bacterium	0.00022
GO:0006596	polyamine biosynthetic process	0.00033
GO:0048544	recognition of pollen	0.00047
GO:0042823	pyridoxal phosphate biosynthetic process	0.00057
GO:0006558	L-phenylalanine metabolic process	0.00068
GO:0006525	arginine metabolic process	0.00068
GO:0008652	cellular amino acid biosynthetic process	0.00107
GO:0051205	protein insertion into membrane	0.00212
GO:0006090	pyruvate metabolic process	0.0026
GO:0009408	response to heat	0.00302
GO:0006099	tricarboxylic acid cycle	0.00332
GO:0006720	isoprenoid metabolic process	0.00459
GO:0046417	chorismate metabolic process	0.00684
GO:0019419	sulfate reduction	0.00684
GO:0034613	cellular protein localization	0.00944
GO:0006413	translational initiation	0.014
GO:0016311	dephosphorylation	0.01514
GO:0006591	ornithine metabolic process	0.0194
GO:0042255	ribosome assembly	0.0194
GO:0006002	fructose 6-phosphate metabolic process	0.02307
GO:0005992	trehalose biosynthetic process	0.02559
GO:0006032	chitin catabolic process	0.02559
GO:0045454	cell redox homeostasis	0.03605
GO:0006222	UMP biosynthetic process	0.03668
GO:0051188	cofactor biosynthetic process	0.04192
GO:0006614	SRP-dependent cotranslational protein targeting to membrane	0.04365
**Molecular Function**		
GO:0030170	pyridoxal phosphate binding	5.50E-06
GO:0043565	sequence-specific DNA binding	1.10E-05
GO:0005509	calcium ion binding	7.50E-05
GO:0046912	transferase activity, transferring acyl groups, acyl groups converted into alkyl on transfer	0.00012
GO:0003700	transcription factor activity, sequence-specific DNA binding transcription factor activity	0.00023
GO:0004842	ubiquitin-protein transferase activity	0.00023
GO:0004385	guanylate kinase activity	0.00041
GO:0016705	oxidoreductase activity, acting on paired donors, with incorporation or reduction of molecular oxygen	0.00049
GO:0004722	protein serine/threonine phosphatase activity	0.00068
GO:0005506	iron ion binding	0.00074
GO:0004298	threonine-type endopeptidase activity	0.00097
GO:0004775	succinate-CoA ligase (ADP-forming) activity	0.00156
GO:0016410	N-acyltransferase activity	0.00288
GO:0003743	translation initiation factor activity	0.00385
GO:0003924	GTPase activity	0.00385
GO:0004222	metalloendopeptidase activity	0.00457
GO:0016597	amino acid binding	0.00529
GO:0004674	protein serine/threonine kinase activity	0.00545

GO:0004158	dihydroorotate oxidase activity	0.00555
GO:0004106	chorismate mutase activity	0.00555
GO:0008898	S-adenosylmethionine-homocysteine S- methyltransferase activity	0.00555
GO:0051082	unfolded protein binding	0.00595
GO:0016841	ammonia-lyase activity	0.00662
GO:0020037	heme binding	0.00664
GO:0016407	acetyltransferase activity	0.00674
GO:0016831	carboxy-lyase activity	0.00774
GO:0051087	chaperone binding	0.00935
GO:0008483	transaminase activity	0.0155
GO:0004549	tRNA-specific ribonuclease activity	0.01582
GO:0004019	adenylosuccinate synthase activity	0.01582
GO:0004617	phosphoglycerate dehydrogenase activity	0.01582
GO:0004568	chitinase activity	0.01696
GO:0030976	thiamine pyrophosphate binding	0.01739
GO:0051536	iron-sulfur cluster binding	0.01894
GO:0000287	magnesium ion binding	0.01949
GO:0015662	ATPase activity, coupled to transmembrane movement of ions, phosphorylative mechanism	0.02064
GO:0016628	oxidoreductase activity, acting on the CH-CH group of donors, NAD or NADP as acceptor	0.02708
GO:0035639	purine ribonucleoside triphosphate binding	0.02927
GO:0004556	alpha-amylase activity	0.03007
GO:0004571	mannosyl-oligosaccharide 1,2-alpha- mannosidase activity	0.03007
GO:0008083	growth factor activity	0.03007
GO:0004664	prephenate dehydratase activity	0.03007
GO:0030151	molybdenum ion binding	0.03007
GO:0009001	serine O-acetyltransferase activity	0.03007
GO:0003872	6-phosphofructokinase activity	0.03331
GO:0016758	transferase activity, transferring hexosyl groups	0.04245
GO:0008312	7S RNA binding	0.04767
GO:0050662	coenzyme binding	0.04789
**RIPE**		
**Biological Process**		
GO:0016567	protein ubiquitination	4.70E-06
GO:0009070	serine family amino acid biosynthetic process	4.70E-05
GO:0050832	defense response to fungus	0.00014
GO:0042742	defense response to bacterium	0.00014
GO:0048544	recognition of pollen	0.00014
GO:0006596	polyamine biosynthetic process	0.00018
GO:0006563	L-serine metabolic process	0.00041
GO:0006558	L-phenylalanine metabolic process	0.00042
GO:0006032	chitin catabolic process	0.00057
GO:0016998	cell wall macromolecule catabolic process	0.0024
GO:0009095	aromatic amino acid family biosynthetic process	0.00285
GO:0006412	translation	0.00603
GO:0006662	glycerol ether metabolic process	0.01136
GO:0009435	NAD biosynthetic process	0.0115
GO:0006536	glutamate metabolic process	0.0115
GO:0055114	oxidation-reduction process	0.01194
GO:0042823	pyridoxal phosphate biosynthetic process	0.01582
GO:0007205	protein kinase C-activating G-protein coupled receptor signaling pathway	0.01678
GO:0008299	isoprenoid biosynthetic process	0.01913
GO:0006816	calcium ion transport	0.02479
GO:0006096	glycolytic process	0.02678
GO:0006820	anion transport	0.03397
GO:0008283	cell proliferation	0.0477
GO:0000103	sulfate assimilation	0.0477
GO:0009873	ethylene-activated signaling pathway	0.0477
GO:0006542	glutamine biosynthetic process	0.0477

GO:0019318	hexose metabolic process	0.04793
**Molecular Function**		
GO:0004674	protein serine/threonine kinase activity	2.90E-08
GO:0048037	cofactor binding	3.00E-07
GO:0003735	structural constituent of ribosome	1.50E-06
GO:0005506	iron ion binding	1.90E-06
GO:0016705	oxidoreductase activity, acting on paired donors, with incorporation or reduction of molecular oxygen	3.90E-06
GO:0005509	calcium ion binding	6.80E-06
GO:0020037	heme binding	1.30E-05
GO:0004842	ubiquitin-protein transferase activity	3.10E-05
GO:0004568	chitinase activity	0.00022
GO:0015662	ATPase activity, coupled to transmembrane movement of ions, phosphorylative mechanism	0.00031
GO:0043565	sequence-specific DNA binding	0.00032
GO:0003700	transcription factor activity, sequence- specific DNA binding transcription factor activity	0.00039
GO:0046912	transferase activity, transferring acyl groups, acyl groups converted into alkyl on transfer	0.00044
GO:0016857	racemase and epimerase activity, acting on carbohydrates and derivatives	0.00045
GO:0016831	carboxy-lyase activity	0.00095
GO:0008061	chitin binding	0.00157
GO:0000287	magnesium ion binding	0.00313
GO:0016841	ammonia-lyase activity	0.00377
GO:0004722	protein serine/threonine phosphatase activity	0.00386
GO:0004781	sulfate adenylyltransferase (ATP) activity	0.00406
GO:0046915	transition metal ion transmembrane trans porter activity	0.00506
GO:0004143	diacylglycerol kinase activity	0.00982
GO:0030060	L-malate dehydrogenase activity	0.01135
GO:0004385	guanylate kinase activity	0.01166
GO:0004124	cysteine synthase activity	0.01166
GO:0008378	galactosyltransferase activity	0.01605
GO:0003951	NAD+ kinase activity	0.02004
GO:0008483	transaminase activity	0.02184
GO:0004402	histone acetyltransferase activity	0.0221
GO:0004556	alpha-amylase activity	0.02234
GO:0004775	succinate-CoA ligase (ADP-forming) activity	0.02234
GO:0008083	growth factor activity	0.02234
GO:0016842	amidine-lyase activity	0.02234
GO:0004664	prephenate dehydratase activity	0.02234
GO:0008977	prephenate dehydrogenase activity	0.03567
GO:0004665	prephenate dehydrogenase (NADP+) activity	0.03567
GO:0008312	7S RNA binding	0.03567
GO:0004866	endopeptidase inhibitor activity	0.0358
GO:0008308	voltage-gated anion channel activity	0.03684
GO:0004743	pyruvate kinase activity	0.03684
GO:0030955	potassium ion binding	0.03684

Supplementary Table 2. Annotation of the top 100 most heterogeneously expressed genes in unripe and ripe fruits in response to *C. scovillei* inoculation.

Gene ID	Annotation	Cluster
CA02g12080	Light harvesting chlorophyll a/b-binding protein	I
CA02g12050	Light harvesting chlorophyll a/b-binding protein	I
CA02g12070	Chlorophyll a/b binding protein	I
CA04g00770	Light harvesting chlorophyll a/b-binding protein	I
CA08g15590	Chloroplast pigment-binding protein CP24	I
CA07g18220	Chlorophyll a/b-binding protein	I
CA09g16970	Unknown protein	I
CA09g16980	Unknown protein	I
CA03g29950	Chlorophyll a/b-binding protein (cab-11)	I
CA04g00760	Light harvesting chlorophyll a/b-binding protein	I
CA07g18550	Unknown protein	I
CA03g00760	Detected protein of unknown function	I
CA10g18910	Cytochrome P450	I
CA02g09470	Cucumisin, putative	I
CA03g22760	UPA16	I
CA07g10990	Chlorophyll a/b-binding protein	I
CA01g34780	Unknown protein	I
CA06g22830	Unknown protein	I
CA07g20940	Photosystem I reaction center subunit V, chloroplastic-like	I
CA05g00450	Unknown protein	I
CA01g08910	Carotenoid cleavage dioxygenase 4	I
CA02g05510	Ribulose bisphosphate carboxylase small chain 2C, chloroplastic-like	I
CA09g16960	Unknown protein	I
CA10g00480	NADPH:protochlorophyllide oxidoreductase	I
CA02g18790	1-aminocyclopropane-1-carboxylate oxidase	I
CA04g12700	Extensin	I
CA02g18250	Secretory peroxidase	I
CA09g14760	21 kDa protein, putative	I
CA10g18210	Tropinone reductase I	I
CA04g00910	MLP-like protein 34-like	I
CA06g12690	Non-specific lipid-transfer protein 2-like	I
CA06g22880	Detected protein of unknown function	I
CA10g10770	Non-specific lipid-transfer protein 2-like	I
CA05g02660	BURP domain-containing protein 17-like	I
CA03g08900	CASP-like protein VIT_07s0104g01350-like	I
CA03g33780	Aspartic proteinase nepenthesin-1, putative	I
CA07g11840	DNA binding protein, putative	I
CA05g08210	Kirola-like	I
CA03g08530	Putative aminotransferase	I
CA09g18170	Zeatin O-glucosyltransferase-like	I
CA07g12580	Xyloglucan endotransglycosylase	I
CA03g30190	42kDa chitin-binding protein	I
CA05g08590	Kirola-like	I
CA07g18560	Major facilitator superfamily	I
CA10g18950	Kunitz-type protease inhibitor KPI-D2.2	I
CA01g10530	Lipoxygenase	I
CA11g05940	Acyltransferase 1	I
CA06g10710	Acyl-[acyl-carrier-protein] desaturase	I
CA07g04400	Tetratricopeptide repeat-like superfamily protein]	I

CA02g00370	Zinc/iron transporter, putative	II
CA05g14280	Probable peroxygenase 5-like	II
CA01g01910	Endoglucanase-like	II
CA01g03620	Green flesh protein	II
CA06g22860	Capsanthin/capsorubin synthase	II
CA10g19580	Polygalacturonase	II
CA08g14620	13S globulin seed storage protein 2-like	II
CA10g14820	Cysteine protease	II
CA03g13030	Mads box protein, putative	II
CA03g13770	Protein phosphatase 2C	II
CA02g00210	Carbonic anhydrase	II
CA01g33610	Detected protein of unknown function	II
CA03g23560	Biotic cell death-associated protein	II
CA10g22130	CYP98A33v1	II
CA05g04830	Multiprotein-bridging factor 1c-like	III
CA05g04810	Zeatin O-glucosyltransferase-like	III
CA09g03110	Putative glutathione S-transferase T2	III
CA01g32430	Transcription factor, putative	III
CA09g03100	Glutathione S-transferase-like protein	III
CA12g04540	Putative pre-mRNA-splicing factor ATP-dependent RNA helicase DHX16-like	III
*CA01g05990**	5-epi-aristolochene 1,3-dihydroxylase	III
CA02g04980	Reticuline oxidase-like protein-like	III
CA02g25720	Patatin-like protein 3	III
CA10g14140	Unknown protein	III
CA10g02550	Cytochrome P450	III
CA04g13070	Pathogen-related protein-like	III
CA02g09540	Premnaspirodiene oxygenase-like	III
*CA02g09520**	5-epi-aristolochene synthase	III
CA11g14520	Cytochrome P450	III
*CA12g05030**	5-epi-aristolochene synthase	III
*CA12g05070**	5-epi-aristolochene 1,3-dihydroxylase	III
CA01g18970	BON1-associated protein 2-like	III
CA04g15690	Unknown protein	III
*CA12g05140**	5-epi-aristolochene 1,3-dihydroxylase	III
CA01g33440	3-hydroxy-3-methylglutaryl coenzyme A synthase	III
*CA02g09570**	5-epi-aristolochene 1,3-dihydroxylase	III
CA05g17820	UTP:alpha-D-glucose-1-phosphate uridylyltransferase	III
CA01g04790	Invertase	III
CA09g03220	Pathogenesis-related leaf protein 4-like	III
CA08g16080	AT3	III
CA05g00170	Unknown protein	III
CA11g02040	RING-H2 finger protein ATL74-like	III
CA08g18080	Allene oxide synthase	III
CA05g18370	Unknown protein	III
CA04g10620	Pepper esterase	III
CA02g15780	Polyphenol oxidase	III
CA07g11250	1-aminocyclopropane-1-carboxylic acid oxidase	III
CA02g04360	Ethylene response factor ERF2	III
CA05g03050	Cytochrome P450 CYP736A54	III
CA03g03950	UDP-sugar:glycosyltransferase	III
CA05g03070	Cytochrome P450 CYP736A54	III

*candidate genes for capsidiol biosynthesis upregulated under *C. scovillei* interaction.

Supplementary Table 3. Primer sequences of candidate genes for capsidiol biosynthesis used in the real time quantitative reactions (RT-qPCR) analyses in pepper fruits.

Gene name	Gene annotation	Gene ID	Accession number	Primer Forward and Reverse	Average efficiency (%)	Amplicon size (bp)
CaEAS	*5-epi-aristolochene synthase*	*CA02g09520*	O65323.1	F- ATGGCCTCAGTTGTAGTTGG<br>R- AAACGATCACCCCATAGACT	110	101
CaEAS	*5-epi-aristolochene synthase*	*CA12g05030*	O65323.1	F- ATGGCCTCAGTTGCAGT<br>R- AACGATCACCCCATAGACT	110	100
CaEAH	*5-epi-aristolochene 1,3-dihydroxylase*	*CA01g05990*	Q94FM7.2	F- AGAATCACCAAACACTCCCA<br>R- CATGGACCTGGAGGCAA	110	132
CaEAH	*5-epi-aristolochene 1,3-dihydroxylase*	*CA12g05070*	Q94FM7.2	F- CTCTCCCAAAATGCAATTCTTC<br>R- GGAGGCAATTTTTTGGTTTGG	110	111
CaUEP	*Ubiquitin extension protein*	*CA12g21050*	DQ975458	F- CCGACTACAACATCCAGAAG<br>R- CACACTCAGCATTAGGACAC	100	207
*CaEF1*α	*Elongation factor 1 alpha*	*CA06g07620*	AY496125	F- TGAAGAATGGTGATGCTGGC<br>R- GACAACACCAACAGCAACAG	100	132
